# Nature-Inspired Pathogen and Cancer Protein Covalent Inhibitors: From Plants and Other Natural Sources to Drug Development

**DOI:** 10.3390/pathogens14111153

**Published:** 2025-11-12

**Authors:** Giovanni N. Roviello

**Affiliations:** Institute of Biostructures and Bioimaging, Italian National Research Council (IBB-CNR), Via P. Castellino 111, 80131 Naples, Italy; giovanni.roviello@cnr.it; Tel.: +39-081220345

**Keywords:** covalent inhibitors, natural products, antimicrobial drug development, pathogen-targeted therapy, plant-derived bioactives, anticancer, phytomolecules

## Abstract

Nature has long served as a prolific source of bioactive compounds, offering structurally diverse scaffolds for the development of therapeutics. In recent years, increasing attention has been given to nature-inspired covalent inhibitors, molecules that form covalent bonds with pathogen- or cancer-specific targets, due to their potential selectivity and sustained biological activity. This review explores the landscape of covalent inhibitors derived from natural sources, with a focus on compounds from fungi, marine organisms, bacteria and plants. In particular, emphasis is placed on the molecular mechanisms through which these compounds exert their activity against different types of pathogens and other biomedically relevant targets, highlighting key structural motifs that facilitate covalent interactions. Furthermore, the review discusses recent advances in synthetic modification, target identification, and optimization strategies that bridge natural compound discovery with modern drug development. By drawing insights from nature’s chemical repertoire, this work ultimately displays the potential of natural covalent inhibitors as a promising foundation for next-generation anti-infective and anticancer therapeutics.

## 1. Introduction

The inhibition of pathogen enzymes [1] has emerged as a powerful and multifaceted therapeutic strategy, offering targeted intervention across a wide spectrum of biological systems and disease contexts. As the global burden of infectious diseases, cancer, and drug-resistant pathogens continues to rise, enzyme inhibition is increasingly recognized as a cornerstone of next-generation antimicrobial, antiviral, and anticancer drug discovery [2,3,4]. This approach enables the disruption of critical molecular processes with high specificity, often reducing the likelihood of resistance development. Enzyme inhibitors are broadly categorized into two mechanistic classes: covalent and non-covalent [5]. Covalent inhibitors form stable chemical bonds with specific amino acid residues, typically nucleophilic side chains such as cysteine or serine, within the active sites of target enzymes [5]. This often results in irreversible or slowly reversible inactivation, leading to prolonged target engagement and potent biological effects. In contrast, non-covalent inhibitors rely on reversible interactions, including hydrogen bonding, ionic forces, van der Waals interactions, and hydrophobic effects, with these transient associations allowing for greater pharmacological flexibility and often improved safety profiles. Understanding the fundamental distinctions between these two classes is essential for rational drug design, as both offer complementary advantages in therapeutic development. Covalent inhibitors, in particular, have garnered renewed interest due to their ability to engage rare or otherwise inaccessible residues within target proteins, enabling the design of highly selective agents [6]. Despite concerns regarding potential toxicity and off-target effects, advances in computational modeling and structural biology have provided deeper mechanistic insights, facilitating the development of safer and more effective covalent therapeutics such as reversible covalent inhibitors [7]. In the context of escalating drug resistance [8], covalent inhibition has demonstrated superior durability and selectivity, reinforcing its relevance in modern pharmacology. Moreover, natural products have long served as a foundational source of drug leads [9,10,11,12,13], offering structurally diverse and biologically active scaffolds. Among these, covalent inhibitors derived from fungi, marine organisms, and microbes have proven especially valuable, as these compounds often contain reactive functional groups, such as Michael acceptors, epoxides, or β-lactam rings, that enable covalent modification of enzyme active sites [14]. In particular, their ability to irreversibly modulate enzymatic activity has translated into therapeutic potential across a range of diseases. On the other hand, plant-derived covalent inhibitors further expand this chemical repertoire [15], with these bioactive molecules frequently featuring electrophilic moieties such as α-exo-methylene lactones, cyclopentenones, isothiocyanates, and epoxides, which facilitate covalent bonding with key protein residues. Covalent inhibitory mechanisms of action are diverse, encompassing inhibition of NF-κB signaling, modulation of redox homeostasis, interference with viral polymerases, and disruption of calcium ATPase activity [16,17,18,19]. The structural complexity and biological potency of these compounds clearly demonstrate their promise as therapeutic agents. This review explores the landscape of nature-inspired covalent inhibitors, with a focus on plant-derived compounds targeting pathogen and cancer-related enzymes. By examining their molecular mechanisms, structural features, and therapeutic applications, we aim to highlight the potential of these natural products as a foundation for the development of next-generation anti-infective and anticancer agents.

### Methodology

**Scheme 1:**
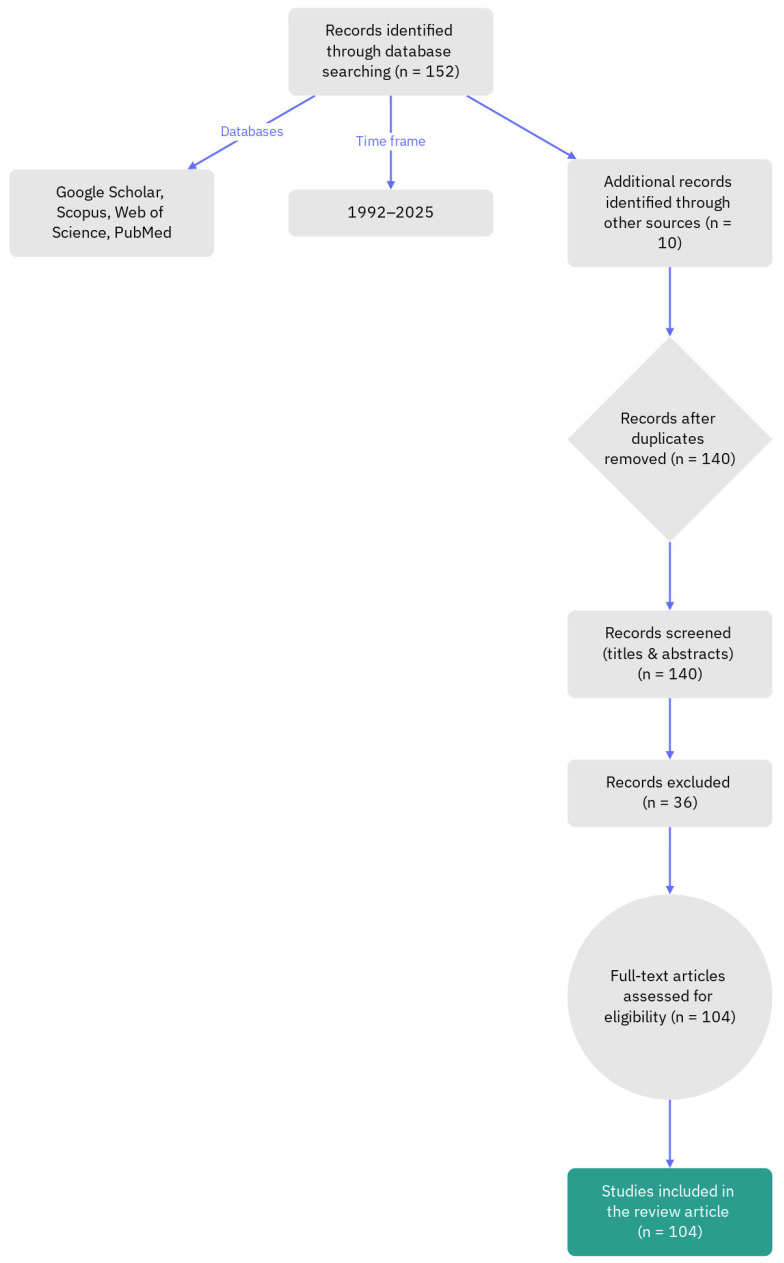
Flowchart representation of the literature review methodology.

This literature review was conducted using a structured and selective approach to identify relevant scholarly publications focused on covalent inhibitors derived from natural sources (Scheme 1).

Only articles published in English were considered for inclusion, while publications in other languages were excluded to maintain consistency and ensure accessibility of the reviewed material. Additionally, duplicated entries and retracted papers were systematically excluded to preserve the integrity and reliability of the data. The research was carried out using all major academic databases and search engines including Google Scholar, Scopus, Web of Science, and PubMed. The time frame of the review spanned from 1992 to 2025, encompassing both foundational and contemporary studies. Of the 104 references reviewed, 70 papers (67%) were published within the last decade (2015–2025), indicating the rapid growth and evolving focus of research in this domain. Search terms were carefully selected and combined using Boolean operators to refine the results. Keywords such as ‘covalent inhibitors’, ‘natural products’, ‘plant-derived’, ‘enzyme inhibition’, and ‘pathogen-targeted’ were used to identify relevant publications. Titles and abstracts were screened for relevance, followed by full-text reviews to assess methodological quality and alignment with the review’s objectives. The final selection of literature provided a robust foundation for analyzing the molecular mechanisms, structural features, and therapeutic applications of nature-inspired covalent inhibitors.

## 2. Covalent Inhibitors from Natural Sources

### 2.1. Inhibition of Pathogen Enzymes as a Therapeutic Strategy

**Figure 1:**
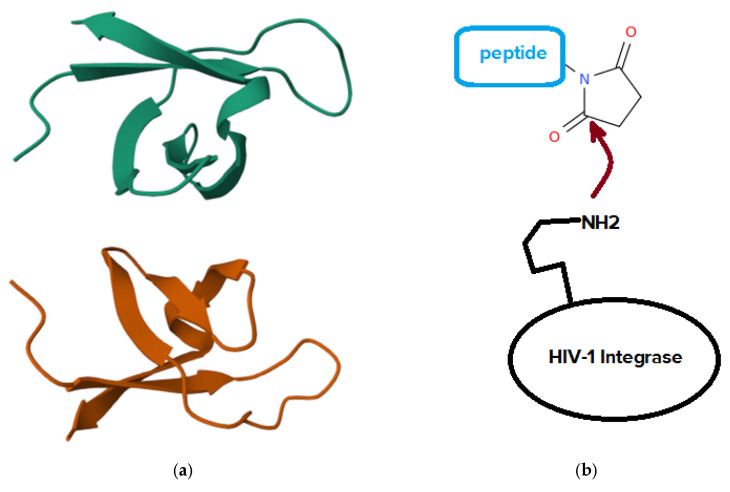
(**a**) Three-dimensional view of the solution structure of the DNA-binding domain of HIV-1 integrase, determined by NMR spectroscopy. The image is adapted from the RCSB Protein Data Bank (PDB ID: 1IHV; https://www.rcsb.org/structure/1IHV, link accessed on 26 October 2025). (**b**) General mechanism for covalent inhibition of HIV-1 integrase by succinimidyl peptides.

The inhibition of pathogen enzymes represents a powerful and multifaceted therapeutic strategy, encompassing diverse biological systems and disease contexts. This approach is increasingly recognized as a cornerstone of next-generation antimicrobial, antiviral, and anticancer drug discovery, due to its capacity to target critical molecular processes with high specificity and reduced risk of resistance. For example, the gastrointestinal tract is among the most exposed systems to proteolytic activity under both normal and pathological conditions [20,21], with evidence indicating that dysregulated protease homeostasis contributes to the pathogenesis of various gastrointestinal disorders. Hence, protease inhibition has shown therapeutic relevance in conditions such as inflammatory bowel diseases, functional gastrointestinal disorders, and colorectal cancer [22]. In fact, proteolytic enzymes perform essential roles in metabolic regulation and biological function, and they are widely utilized in biotechnology, and due to the critical nature of their activity, a precise regulation of these enzymes is required. Thus, molecules that inhibit proteases serve as versatile tools in medicine applications. In medical contexts, protease inhibitors are employed as diagnostic and therapeutic agents for viral, bacterial, fungal, and parasitic infections, as well as for cancer, immunological disorders, neurodegenerative conditions, and cardiovascular diseases. Moreover, they contribute to crop protection against plant pathogens and herbivorous pests [23], and help mitigate abiotic stress such as drought [24]. In laboratory settings, protease inhibitors are used to prevent unwanted proteolysis during protein extraction or heterologous expression. The vast diversity of proteases found in prokaryotes, yeasts, filamentous fungi, and mushrooms suggests that these organisms may also serve as rich sources of novel protease inhibitors [25]. Antibiotic resistance, including multidrug resistance, presents a major challenge to public health by diminishing the effectiveness of conventional antimicrobial therapies. Therefore, many existing antibiotics fail to produce adequate therapeutic outcomes against resistant bacterial strains. In response to widespread antibiotic inefficacy, enzymatic therapy, particularly in combinatorial formats, has emerged as a promising strategy for managing bacterial infections and addressing resistance. Combinatorial enzymatic approaches offer multiple beneficial properties: strong antibacterial performance, biofilm disruption, immunomodulatory effects, targeted and synergistic mechanisms, multi-site action, and reduced susceptibility to resistance development, with these characteristics positioning enzymatic combinations as viable alternatives or complements to traditional antibiotic treatments in the effort to combat antimicrobial resistance [26]. Famously, clinically relevant antibiotic resistance [27] has emerged against nearly all antibiotics currently in use. However, the development of new antibiotic classes has not kept pace with the increasing demand for effective treatments. Instead of relying solely on agents that inhibit bacterial viability in vitro, alternative strategies focus on targeting functions critical to infection, such as virulence factors responsible for host damage and disease progression. In fact, this approach offers several advantages: it broadens the spectrum of bacterial targets, helps preserve the host’s endogenous microbiota, and applies reduced selective pressure, which may contribute to lowering the risk of resistance development [28]. Inhibiting pathology-related enzymes is crucial also in the therapy of neurodegenerations. In fact, in the context of neurodrug discovery, the amyloid cascade hypothesis has led to the identification of several enzymatic targets with therapeutic relevance for Alzheimer’s disease [29], among which α secretase, β secretase, and γ secretase are particularly notable due to their involvement in the proteolytic processing of amyloid precursor protein and their central role in the generation and regulation of amyloid-β peptides. These enzymes are susceptible to modulation by small molecules in both experimental and clinical contexts, although their participation in other essential physiological pathways introduces a considerable risk of mechanism-related toxicity when their activity is inhibited. Addressing this challenge requires careful delineation of the therapeutic window and refinement of drug selectivity to ensure targeted modulation of amyloid precursor protein processing while minimizing off-target effects. In the same context, secretase inhibitors are anticipated to be among the earliest small-molecule candidates undergoing comprehensive clinical evaluation for disease-modifying potential in Alzheimer’s disease, and the outcomes of these initial trials are expected to significantly influence future pharmacological strategies not only for Alzheimer’s disease but also for a broader spectrum of neurodegenerative disorders [30]. Another biomedically related topic is the development of antiviral strategies and in this frame, HIV-1 integrase [31] (Figure 1), a protein with a molecular weight of approximately 32,000 Da essential for HIV virus life cycle, constitutes a valid target for anti-HIV drugs.

In fact, the integration of HIV DNA into the host cell genome proceeds through a defined sequence of molecular events, beginning with 3′ processing of the viral DNA and followed by strand transfer into the host chromosomal DNA, with this integration step marking the biochemical culmination of HIV entry into human cells, such as T lymphocytes. In contrast to the substantial progress achieved in developing clinically approved inhibitors targeting HIV reverse transcriptase and HIV protease, not many drugs have received the due attention for clinical use based on inhibition of HIV integrase. Advances in the development of integrase inhibitors have highlighted several promising compound classes, including diketo acids bearing aromatic or heteroaromatic groups, diketo acids with nucleobase frameworks, bis-diketo acid derivatives, functionalized naphthyridines, and other structural analogs of diketo acids. Experimental data on integrase inhibition and in vitro anti-HIV activity have contributed to the characterization of these compounds. Several agents have entered clinical trials, with earlier candidates such as S-1360, L-870,810, and L-870,812, and more recent compounds including GS-9137 and MK-0518 undergoing evaluation for therapeutic potential [32]. An example of covalent inhibitors of HIV-1 integrase is provided by N-succinimidyl peptides that take advantage of the direct binding of the succinimide to a lysine of the integrase [33]. Overall, the inhibition of pathogen enzymes offers a versatile and promising therapeutic avenue across multiple domains of human health, and biotechnology. While this section highlights only some of the numerous examples available, the topic is vast, encompassing a wide array of biological mechanisms, enzyme classes, and application areas.

### 2.2. Difference Between Covalent and Non-Covalent Enzyme Inhibition

Enzyme inhibition can be broadly classified into two principal categories: covalent and non-covalent inhibition (Table 1) [34].

**Figure 2:**
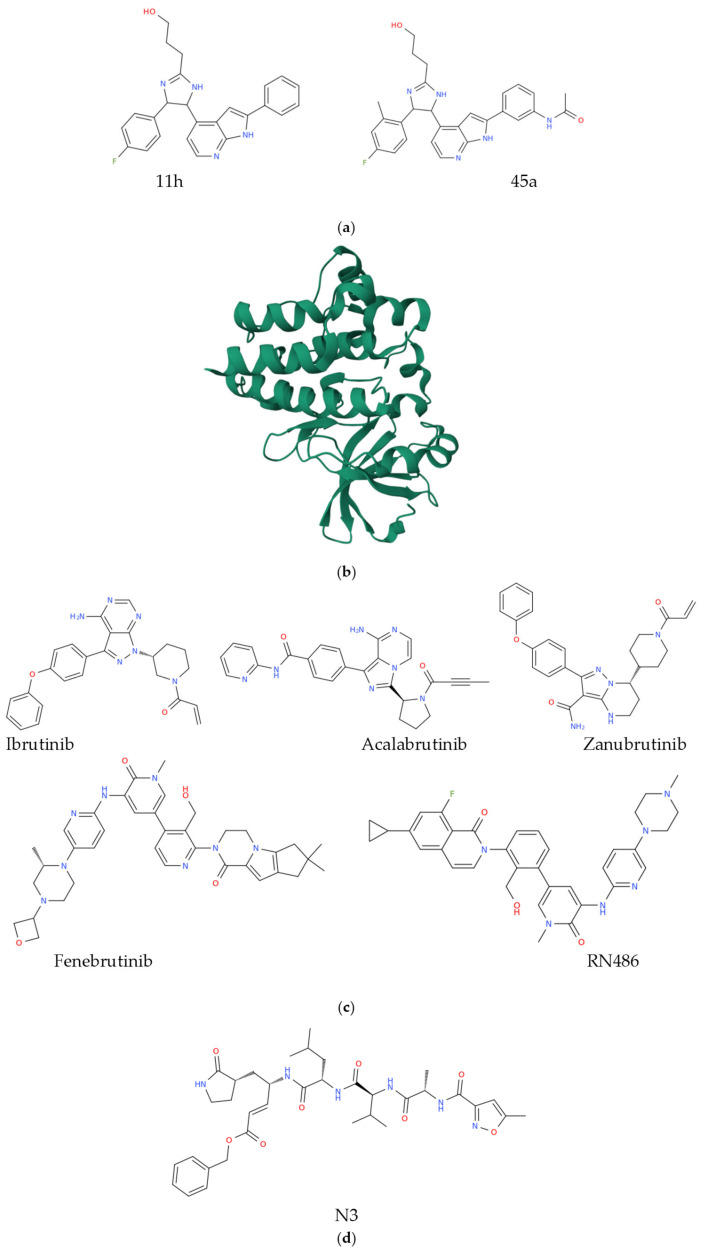
Chemical representation of compounds 11h and 45a (**a**). Three-dimensional structure of unliganded Bruton tyrosine kinase (**b**), (https://www.rcsb.org/3d-view/6S90/1 link accessed on 26 October 2025; PDB ID 6S90). Structural representation of the inhibitors of the Bruton tyrosine kinase mentioned in this section (**c**) and of the Mpro inhibitor N3 (**d**).

Covalent inhibition involves the formation of a stable chemical bond between the inhibitor and the target enzyme, often resulting in irreversible or slowly reversible inactivation, with the inhibitors belonging to this family typically interacting with specific amino acid residues, forming covalent linkages that block the enzyme’s catalytic function. In contrast, non-covalent inhibitors bind to enzymes through reversible interactions such as hydrogen bonding, ionic forces, van der Waals interactions, or hydrophobic effects, allowing for transient association and dissociation under physiological conditions. While covalent inhibitors generally exhibit prolonged target engagement and high potency, non-covalent inhibitors often offer improved safety profiles and tunable pharmacodynamics. Understanding the fundamental differences between these two classes of enzyme inhibitors is critical for rational drug design, as both approaches play complementary roles in modern therapeutic development. The pace and effectiveness of strategies for drug target identification continue to generate discussion within the field of pharmaceutical development. Importantly, covalent inhibitors offer notable advantages compared to their non-covalent counterparts, particularly due to their ability to engage rare amino acid residues within target proteins, which facilitates the creation of highly selective therapeutic agents. Despite these benefits, concerns regarding toxicity remain a significant challenge for this class of compounds, and issues such as irreversible drug-induced toxicity and reduced reactivity in reversible systems have prompted further investigation, including computational analyses that provide mechanistic insights. Remarkably, both covalent and non-covalent inhibitors present distinct advantages; however, in the context of rising drug resistance, covalent inhibition has demonstrated greater suitability and will be more extensively described in this work. The enhanced selectivity observed in covalent systems supports their growing application as therapeutic regimens across diverse clinical settings, and their continued evaluation may inform the selection of lead compounds in future drug discovery efforts [35]. The 20S proteasome core particle [36] represents the proteolytically active component of the ubiquitin-proteasome system, which governs the majority of intracellular protein degradation in eukaryotic cells. Its therapeutic relevance has gained prominence following the regulatory approval of bortezomib, a first-in-class compound targeting this system for anticancer treatment [37], that, along with other second-generation inhibitors currently under clinical investigation, typically forms covalent and irreversible interactions with the proteolytic sites of the core particle, resulting in sustained inhibition. These compounds often contain reactive functional groups that contribute to nonspecific binding at proteasomal active centers and lead to considerable off-target enzymatic effects, which are associated with adverse side effects. Building on this, reversible proteasome covalent inhibitors [38,39] have been proposed as a potential alternative to address these limitations, although their development requires careful optimization of enthalpic and entropic parameters to achieve effective and selective binding. Structural insights derived from crystallographic studies have contributed to a deeper understanding of reversible inhibition mechanisms and may inform future strategies in proteasome-targeted drug design [38]. A major obstacle in the therapeutic management of non-small cell lung cancer involving epidermal growth factor receptor is the emergence of drug resistance driven by somatic mutations. Among these, the L858R and T790M double mutant variant has been identified as particularly challenging due to its reduced susceptibility to existing inhibitors. Although numerous compounds have been developed, most have failed to effectively inhibit this mutant form. Recent investigations have identified two molecules, 11h and 45a (Figure 2a), as promising inhibitors that operate through distinct mechanisms involving noncovalent and covalent interactions, respectively. Despite their potential, the structural and dynamic consequences of these binding modes have not been fully characterized. On the other hand, molecular dynamics simulations combined with free energy calculations have provided atomistic insights into the nature of these interactions, revealing a substantial difference in binding free energy between the two compounds, with 45a exhibiting a more favorable profile. Also, van der Waals forces were found to be the dominant contributors to binding in both cases, with a stronger influence observed for 45a. Specific residues, including ARG841 and THR854, were shown to play a stabilizing role in the interaction between 45a and the mutant receptor by anchoring its flexible alcohol chain. Furthermore, binding of 45a induces conformational changes within the active site, facilitating improved access to the target residue CYS797 [40]. Low molecular weight compounds have played a central role in drug development due to their pharmacological versatility. Despite their widespread use, challenges related to target specificity and binding efficiency continue to limit their clinical applicability. As mentioned above, protein kinases have emerged as attractive therapeutic targets, although their structural conservation and large family size present significant obstacles to selective inhibition. Among these, Bruton tyrosine kinase (Figure 2b) [41], a cytoplasmic member of the tyrosine kinase family, has gained considerable attention for its therapeutic potential in B-cell malignancies and, more recently, in autoimmune and inflammatory conditions. Structural characterization and binding mechanisms of small molecule inhibitors targeting Bruton tyrosine kinase have revealed two principal classes: irreversible inhibitors, including ibrutinib, acalabrutinib, and zanubrutinib [42], and reversible inhibitors such as fenebrutinib and RN486 [43] (Figure 2c).

These compounds exhibit distinct interaction profiles with the kinase domain, and their structure–function relationships continue to inform the design of next-generation therapeutics with improved selectivity and safety profiles [43]. COVID-19, the disease caused by the coronavirus SARS-CoV-2 [44], has triggered a global crisis affecting health systems, economies, and societies worldwide [45]. Therapeutic options for both treatment and prevention remain limited, drawing attention to the urgency of identifying molecular targets that can support the development of safe and effective antiviral agents, particularly in light of the persistent challenge posed by evolving SARS-CoV-2 mutations. The viral main protease, known as Mpro [46], has emerged as a promising target due to its essential role in the proteolytic processing of viral polyproteins and the absence of homologous enzymes in humans, which reduces the likelihood of off-target effects. High-resolution crystallographic structures of Mpro bound to various inhibitors have recently been elucidated, offering valuable insights for structure-guided drug design. A central objective in this approach is the accurate prediction of receptor-ligand binding affinities to inform rational drug discovery. Computational analyses, including alchemical absolute binding free energy and quantum mechanics/molecular mechanics calculations, have been employed to evaluate the total binding energy of two covalent inhibitors of Mpro, specifically N3 and the α-ketoamide inhibitor. These energies comprise both covalent and noncovalent contributions, which differ markedly between the two compounds. The N3 inhibitor (Figure 2d) demonstrates stronger noncovalent interactions, particularly through hydrogen bonding, within the Mpro binding site compared to the α-ketoamide derivative. Additionally, the Gibbs energy of reaction for the formation of the Mpro–N3 covalent complex is higher than that of the Mpro–α-ketoamide complex. These variations in binding energetics may account for observed differences in antiviral activity under experimental conditions and these in silico results align with the reversible and irreversible nature of the respective inhibitors, also emphasizing the significance of both covalent and noncovalent interactions in determining the absolute binding affinity of covalent inhibitors, offering guidance for the design and optimization of targeted therapeutics against SARS-CoV-2 [47]. In summary, covalent and non-covalent enzyme inhibition represent two fundamentally distinct yet complementary mechanisms that underpin much of modern drug discovery. Covalent inhibitors, through the formation of stable chemical bonds, offer strong and often prolonged target engagement but may present toxicity or off-target risks. Non-covalent inhibitors, conversely, rely on transient interactions that afford greater reversibility, safety, and flexibility in pharmacological modulation. The examples presented herein illustrate only some of the numerous cases where these mechanisms have been applied, yet the topic is vast, spanning diverse therapeutic areas.

### 2.3. Covalent Inhibitors from Fungi, Marine Organisms, and Microbes

Natural products have long served as valuable molecular frameworks for drug development [48], and numerous derivatives have been identified as inhibitors of protein tyrosine phosphatases associated with disease [49]. In this context, covalent inhibitors derived from fungi, marine organisms, and microbes have emerged as powerful tools in drug discovery due to their ability to form irreversible interactions with target proteins (Table 2).

These compounds often exploit reactive functional groups, such as Michael acceptors [50], epoxides, or β-lactam rings, to covalently modify nucleophilic residues, most commonly cysteine or serine, within enzyme active sites. One such compound, 4-isoavenaciolide, isolated from a fungal source, has been characterized as an irreversible inhibitor of the dual-specificity phosphatase VHR, with a reported half-maximal inhibitory concentration of 1.4 micromolar [51]. Mass spectrometric analysis confirmed the covalent modification of VHR by two molecules of 4-isoavenaciolide at distinct sites, specifically cysteine 124 within the catalytic domain and cysteine 171 located on the α6 helix of the surface domain. The presence of a reactive exo methylene group in the compound supports a proposed mechanism involving Michael addition to the thiol group of the catalytic cysteine. Notably, 4-isoavenaciolide exhibited selective inhibition of dual-specificity and tyrosine phosphatases, without affecting serine/threonine phosphatases such as PP1 and PP2A. These findings suggest that 4-isoavenaciolide functions as a cysteine-directed inhibitor targeting phosphatases that share the conserved HCX5RS or HCX5RT motif within their active sites [51]. Resorcylic acid lactones constitute a diverse group of polyketide-derived natural products synthesized by various fungal species and have demonstrated potential as ATP-competitive kinase inhibitors [52]. The resorcylic acid moiety within these compounds is capable of mimicking the critical hydrogen-bonding interactions formed by the purine ring of ATP at the nucleotide binding site of kinases. Hypothemycin [53], which contains a cis enone group embedded in its macrocyclic structure, has been shown to selectively interact with a subset of kinases that share a conserved cysteine residue, with notable specificity for cysteine 164 in ERK2 [52]. Structural analogs related to radicicol, a noncovalent inhibitor of HSP90 despite possessing an electrophilic group, and hypothemycin derivatives have contributed to the identification of inhibitors targeting VEGFR and PDGFR with demonstrated in vivo efficacy. More recently, hypothemycin has been reported to exhibit therapeutic activity against sleeping sickness caused by *Trypanosoma brucei* with proteomic analyses revealing that hypothemycin covalently inactivates kinases containing a CDXG motif, including TbGSK3short and TbCLK1, the latter being previously uncharacterized and responsible for the compound’s cytotoxicity against the parasite in vitro and in infected animal models [52]. Pyranonaphthoquinone lactones [54], including 7-deoxykalafungin, lactoquinomycin, and frenolicin B, have been identified as selective and potent inhibitors of Akt, a kinase frequently dysregulated in cancer, diabetes, and neurodegenerative disorders, with half-maximal inhibitory concentrations ranging from 150 to 200 nM. These compounds covalently bind to cysteine 310 in Akt with high selectivity over other members of the AGC kinase family, many of which also possess this conserved residue. The structural features of pyranonaphthoquinone lactones do not readily reveal their binding mechanisms, but their activation as Michael acceptors has been attributed to a thiol-mediated reduction of the quinone group, accompanied by lactone elimination and formation of a reactive quinone methide intermediate. Investigations into the structure-activity relationships of 7-deoxykalafungin have confirmed the essential role of both the quinone and lactone moieties in Akt inhibition, while removal of the pyran ring enabled covalent targeting of cysteine 199 in PKAα. This activation mechanism offers valuable insights for the rational design of chemical probes and therapeutic agents incorporating masked Michael acceptors [52]. Ophiobolin A [55], a phytotoxic compound isolated from fungi belonging to the *Bipolaris* genus (Table 2), exhibits cytotoxic properties through parapoptotic mechanisms at nanomolar concentrations across various cancer cell lines, including glioblastoma, and has demonstrated antitumor efficacy reflected in prolonged mouse survival when tested at 10 mg/kg in the B16F10 mouse melanoma model featuring lung pseudometastases [52]. Its bioactivity is attributed to the presence of a 1,4-dicarbonyl functional group, which undergoes Paal-Knorr pyrrole formation [56] with primary amines, suggesting the potential for covalent modification of lysine residues in an unidentified protein target. To elucidate its mechanism of action, loss-of-function mutants of the KBM7 cell line were employed, revealing that disruption of the phosphatidylethanolamine biosynthetic pathway resulted in diminished cellular response to ophiobolin A. This finding led to the identification of phosphatidylethanolamine as the molecular target. Importantly, the formation of a covalent adduct between ophiobolin A and phosphatidylethanolamine was shown to compromise model membrane integrity, indicating that membrane disruption may underlie its cytotoxic effects in cancer cells [52]. Marine-derived natural products constitute other valuable sources of covalent inhibitors with biomedical relevance. Among these, the fimbrolide compounds [57] isolated from the red algae *Delisea pulchra* exhibit structurally distinctive features, notably their polybrominated butenolide scaffolds. These molecules were initially recognized for their ability to interfere with quorum sensing in bioluminescent Gram-negative *Vibrio* species and have since demonstrated broader antibacterial activity against other pathogenic strains including *Pseudomonas aeruginosa* and *Staphylococcus aureus* [52]. The presence of bromoolefin functionalities renders these compounds particularly reactive, facilitating covalent bond formation with nucleophilic residues in proteins through an addition–elimination mechanism that minimizes the likelihood of reversibility. Experimental studies confirmed that fimbrolides covalently modify LuxS [58,59], a key enzyme in quorum sensing, with early hypotheses suggesting interaction at a non-catalytic cysteine residue. To refine this understanding, a series of alkyne-tagged fimbrolide analogs was synthesized and applied in competitive activity-based protein profiling combined with quantitative mass spectrometry. This approach verified LuxS as a covalent target and identified cysteine 83, located within the catalytic domain, as the actual site of modification rather than the previously proposed cysteine 128. Additional targets were uncovered, including LuxE [60], which undergoes covalent modification at catalytic cysteine 362, and two further proteins: a CBS domain-containing inosine monophosphate dehydrogenase-related protein and PhaB [61], an enzyme involved in polyhydroxybutyrate biosynthesis [62]. These findings not only expand the repertoire of quorum sensing modulators but also highlight the mechanistic advantages of the addition–elimination pathway, which offers enhanced stability of covalent adducts and supports the development of irreversible inhibitors with high target specificity [52]. Famously, bacterial sources have long contributed to the development of covalent inhibitors with therapeutic utility, exemplified by β-lactam antibiotics such carbapenems [63,64], many of which are produced by *Streptomyces* bacteria [65], which remain effective agents for treating bacterial infections. Since the initial discovery of penicillin G nearly a century ago, a natural product of fungi, specifically from *Penicillium* species (like *Penicillium chrysogenum*) [66], this class has expanded to encompass more than fifty clinically approved compounds. All β-lactam antibiotics exert their antibacterial effects by targeting penicillin-binding proteins, a group of dd-transpeptidases [67] essential for bacterial cell wall biosynthesis. The defining feature of these molecules is the four-membered β-lactam ring, which is highly strained and therefore susceptible to nucleophilic attack, enabling the formation of a covalent acyl adduct with the catalytic serine residue located within the penicillin-binding domain of these enzymes. This covalent modification irreversibly inhibits transpeptidase activity, ultimately leading to cell wall disruption and bacterial lysis [68]. Despite their efficacy, many β-lactam antibiotics are vulnerable to hydrolysis by bacterial β-lactamases, which cleave the β-lactam ring and render the compounds inactive. Carbapenems, however, exhibit resistance to hydrolysis by several serine-based β-lactamases, including those in classes A, C, and D, largely due to the nature of their C6 lateral chain in combination with its stereochemistry. To enhance the resistance profile of penicillin derivatives, a modified compound known as MPC-1 was also developed by incorporating carbapenem-like stereochemical features into the penicillin scaffold. These modifications include a trans arrangement of hydrogen atoms at positions C-5 and C-6, as well as substitution of the original 6-β aminoacyl group with a 6-α hydroxyethyl moiety. MPC-1 selectively inhibits class C β-lactamases such as P99 by forming a stable, nonhydrolyzable acyl adduct, and exhibits inhibitory potency two to five times greater than that of clinically used β-lactamase inhibitors such as clavulanate and sulbactam [68]. Structural analysis of the MPC-1–P99 complex revealed a unique binding mode that closely resembles the interaction of carbapenems with class A β-lactamases. In this configuration, the carboxyl group of MPC-1 obstructs the catalytic water molecule required for deacylation, thereby stabilizing the covalent adduct and preventing hydrolytic turnover. These findings suggest that by adopting carbapenem-like stereochemistry, existing penicillin and cephalosporin (from fungi *Acremonium*, formerly *Cephalosporium* [69,70]) frameworks may be reengineered into promising candidates for the development of next-generation β-lactamase inhibitors with enhanced stability and specificity [68]. Clavulanic acid [71,72], a β-lactam compound biosynthesized by the bacterium *Streptomyces clavuligerus* [73], functions as a suicide substrate for serine β-lactamases by covalently modifying the catalytic serine residue within the enzyme’s active site. This irreversible inhibition mechanism protects co-administered β-lactam antibiotics from enzymatic degradation, thereby enhancing their antibacterial efficacy. Although clavulanic acid itself lacks intrinsic antibiotic activity, it has been clinically utilized since the late 1970s in combination therapies, most notably with amoxicillin, and is generally well tolerated with a safety profile characterized by predominantly mild adverse reactions [74]. Beyond its established role in antimicrobial therapy, clavulanic acid has attracted interest for its potential effects on the central nervous system. In 2005, it was reported that β-lactam compounds can upregulate the expression of GLT-1, a glutamate transporter critical for maintaining synaptic glutamate homeostasis and implicated in various neurological disorders [75]. This observation, combined with favorable pharmacokinetic properties, led to a series of preclinical investigations evaluating the neuroactive potential of clavulanic acid. Experimental studies demonstrated that clavulanic acid enhances GLT-1 expression in key brain regions such as the nucleus accumbens, medial prefrontal cortex, and spinal cord, influencing both glutamatergic and dopaminergic neurotransmission. Additionally, it was shown to exert anti-inflammatory effects by modulating cytokine levels, including tumor necrosis factor alpha and interleukin 10 [74]. These neurobiological actions have been explored in a range of preclinical models, where clavulanic acid exhibited beneficial outcomes in conditions such as epilepsy, addiction, stroke, neuropathic and inflammatory pain, dementia, Parkinson’s disease, and behavioral alterations related to anxiety and sexual function. The convergence of neuroprotective, anti-inflammatory, and neurotransmission-modulating properties positions clavulanic acid as a promising candidate for therapeutic repurposing in central nervous system disorders. Continued investigation into its mechanisms and efficacy across diverse neurological models may support its transition from antimicrobial adjunct to multifunctional neurotherapeutic agent [74]. Fosfomycin [76], initially referred to as phosphonomycin, is a broad-spectrum antibiotic originally identified in fermentation broths of *Streptomyces fradiae* [77] through a collaborative effort in Spain involving Merck and the Compañía Española de Penicilina y Antibióticos [78]. Its development began in Europe during the early 1970s, first as an intravenous formulation of the disodium salt and later as an oral preparation in the form of fosfomycin trometamol. The compound has been widely used in countries such as Spain, Germany, France, Japan, Brazil, and South Africa, primarily for the oral treatment of urinary tract infections, although its application has extended to other clinical indications. In the United States, fosfomycin tromethamine was approved in 1996 for single-dose oral therapy targeting uncomplicated urinary tract infections caused by *Escherichia coli* and *Enterococcus faecalis*. Due to the growing challenge of antibiotic resistance, fosfomycin has gained renewed interest for its activity against multidrug-resistant pathogens, and its parenteral use is currently under development for the treatment of complicated urinary tract infections [78]. Mechanistically, fosfomycin acts by inhibiting the MurA enzyme, UDP-N-acetylglucosamine-enolpyruvyltransferase, which catalyzes the initial committed step in bacterial peptidoglycan biosynthesis [78]. This enzymatic reaction involves the transfer of an enolpyruvyl group from phosphoenolpyruvate to UDP-N-acetylglucosamine, forming UDP-GlcNAc-enoylpyruvate and inorganic phosphate. Competitive inhibition studies revealed that fosfomycin mimics phosphoenolpyruvate, functioning as a structural analog. The compound undergoes a time-dependent covalent interaction with MurA, specifically through nucleophilic attack by cysteine 115 on the epoxide ring of fosfomycin. This covalent modification effectively blocks the catalytic activity of MurA, thereby halting cell wall synthesis and exerting bactericidal effects. Remarkably, cysteine 115 is recognized as the critical residue responsible for the enzymatic transformation of phosphoenolpyruvate, and its irreversible engagement by fosfomycin demonstrates the compound’s utility as a covalent inhibitor in antimicrobial therapy [78]. Menadione (Table 2), also known as vitamin K3, is a naphthoquinone derivative produced by bacteria [79] and prenylated by the gut microbiota to form menaquinones (vitamin K_2_) [80,81,82], that has demonstrated antiproliferative effects [83] across multiple human cell lines. Compared to other quinone-based chemotherapeutic agents, this compound exhibits reduced toxicity, enhancing its therapeutic appeal. Its mechanism of action involves covalent modification of the active site of CDC25A, a cell cycle regulatory phosphatase. Building on this observation, a series of hydrophilic naphthoquinone analogs was screened for inhibitory activity against CDC25A. Among the tested compounds, one derivative bearing multiple hydroxyl and halogen substituents displayed the highest potency. Functional assays using flow cytometry confirmed that treatment with this analog resulted in a delay in cell cycle progression at the G1 to S phase transition, consistent with reduced CDC25A activity. These findings support the potential of structurally optimized naphthoquinone derivatives as covalent inhibitors of CDC25A and highlight their relevance in modulating cell cycle dynamics for therapeutic applications [51].

In other words, natural products from fungi, marine organisms, and microbes continue to provide a rich source of covalent inhibitors with highly specific and potent biological activities. By targeting key nucleophilic residues in proteins, these compounds achieve irreversible modulation of enzymatic activity, which translates into therapeutic potential against infectious diseases, cancer, and other pathologies. The mechanistic insights gained from studies of fungal metabolites, marine fimbrolides, and microbial antibiotics demonstrate the value of covalent inhibition strategies in drug development. Furthermore, the rational design and structural optimization of these natural scaffolds offer promising avenues for the creation of next-generation covalent therapeutics with enhanced selectivity, stability, and efficacy.

### 2.4. Covalent Inhibitors from Plants

Plant-derived covalent inhibitors represent a diverse class of bioactive natural products capable of forming irreversible or reversible covalent bonds with specific protein residues (Table 3), predominantly cysteine.

These compounds often possess electrophilic moieties such as α-exo-methylene lactones, epoxides, cyclopentenones, or isothiocyanates, enabling targeted modulation of enzymes, transcription factors, or signaling proteins. Their mechanisms of action include inhibition of NF-κB signaling, modulation of redox homeostasis, interference with viral polymerases, and disruption of calcium ATPase activity. Isothiocyanates are naturally occurring electrophilic compounds found in various foods, particularly as metabolic derivatives of glucosinolates in cruciferous vegetables. Allyl isothiocyanate, a representative member of this class, is responsible for the pungent flavor of mustard and wasabi, an effect linked to activation of the TRPA1 ion channel through covalent interactions with cysteine and lysine residues. Several isothiocyanates, including phenethyl isothiocyanate and sulforaphane, have been evaluated in clinical trials across all phases for diverse therapeutic applications such as cancer chemoprevention and treatment of neurological disorders including schizophrenia and autism spectrum conditions [84]. Their widespread presence in dietary sources and clinical tolerability suggest a favorable safety profile, although their biological effects are often attributed to interactions with multiple molecular targets. These compounds readily undergo conjugation with glutathione to form dithiocarbamates, a reversible process that can lead to trans-thiocarbamoylation of proteins. While their reactivity with amino groups to form stable thioureas is relatively low, lysine residues may still be modified through equilibrium-driven or direct reaction pathways. The electrophilic character of isothiocyanates can be modulated by substituents, with electron-withdrawing groups enhancing reactivity and electron-donating groups diminishing it [84]. Covalent labeling of cysteine residues has been observed in several proteins, including glutathione S-transferase pi, Keap1, protein tyrosine phosphatases, and MEKK1. Interaction with Cys347 in tubulin has been proposed as a key mechanism underlying their biological activity, and modification of the N-terminal catalytic proline in macrophage migration inhibitory factor has also been reported. The reversible nature of cysteine binding complicates target identification in living systems, and despite extensive research over several decades, systematic investigations into structure-activity relationships and applications in targeted covalent inhibitor design remain limited, likely due to the complex equilibria governing isothiocyanate behavior and bioavailability in vivo [84]. A set of ten phytochemicals, previously isolated and characterized from medicinal plants native to Saudi Arabia, was evaluated for their potential to inhibit the viral RNA-dependent RNA polymerase of SARS-CoV-2. Computational studies involving noncovalent docking revealed that all ten compounds formed hydrogen bonds with template primer nucleotides, specifically adenine and uracil, as well as with key residues within the active site of the enzyme, including ASP623, LYS545, ARG555, ASN691, SER682, and ARG553. Further analysis through covalent docking indicated that three of the examined compounds exhibited irreversible binding interactions with CYS813, a critical residue located in the palm domain of the polymerase. Molecular dynamics simulations confirmed the structural stability of the enzyme-inhibitor complexes formed by these three compounds. Therefore, these findings highlight the potential of the three phytocompounds as non-nucleoside inhibitors with irreversible binding characteristics, offering promising scaffolds for the development of targeted antiviral agents against SARS-CoV-2 [85]. Parthenolide [86], a sesquiterpene lactone recognized as the principal bioactive constituent of Feverfew (*Tanacetum parthenium*) extracts, has attracted considerable attention due to its pharmacological properties [52]. It has been identified as an inhibitor of the NF-κB signaling cascade, which regulates the transcription of genes implicated in inflammation and oncogenesis. Although initial findings suggested that parthenolide modulates this pathway upstream of the NF-κB inhibitor protein IκBα, the precise molecular target remained unclear. Subsequent studies employing both native parthenolide and a biotin-conjugated analog demonstrated inhibition of TNFα-induced NF-κB activation, with electrophoretic mobility shift assays revealing covalent interaction with IKKβ, the kinase responsible for IκBα phosphorylation [52]. The compound contains two electrophilic groups, an α-exo-methylene-γ-butyrolactone and an epoxide, and reduction of the α-exo-methylene moiety abolished its anti-inflammatory activity without affecting probe binding, thereby confirming the covalent mechanism. Mutation of cysteine 179 in IKKβ resulted in complete loss of biological activity, indicating this residue as the site of covalent engagement. Due to limitations in solubility and bioavailability, a prodrug derivative known as LC-1 was developed, incorporating an N,N-dimethylamino group that significantly enhanced aqueous solubility and is currently under investigation for applications in acute myelogenous leukemia and drug-resistant glioblastoma [52]. Deoxyelephantopin, another sesquiterpene lactone with a highly functionalized germacranolide framework, is the major active component of *Elephantopus scaber* extracts used in traditional medicine. This compound demonstrated superior efficacy to taxol in suppressing orthotopic breast tumors in murine models and was also shown to inhibit proteasome function and NF-κB signaling. Surface plasmon resonance studies suggested reversible binding to the nuclear receptor PPARγ within its ligand-binding domain, which contains a cysteine residue known to interact covalently with oxidized fatty acids. However, the experimental timeframe may have been insufficient to detect covalent bond formation. A synthetic campaign yielded a library of analogs, including alkyne-tagged variants, and cytotoxicity was attributed to caspase-dependent apoptosis. Proteomic profiling identified eleven covalent targets, including CBS, CTTN, and CSTB, whose depletion is associated with cell death. Further analysis revealed that deoxyelephantopin acts as a covalent antagonist of the nuclear receptor PPARγ [87,88] by engaging cysteine 176 within the zinc-finger domain, rather than the anticipated ligand-binding site, thereby disrupting high-affinity DNA interactions. A more potent binder was also identified from the analog library, marking the first instance of a covalent inhibitor targeting cysteine residues in a zinc-finger domain, which may offer enhanced specificity by acting downstream in the signaling pathway [52]. Helenalin [89], a pseudo-guaianolide found in *Arnica* and *Helenium* species (Table 3), is another sesquiterpene lactone with notable biological activity [52]. It contains both an α-exo-methylene-γ-butyrolactone and a cyclopentenone, which contribute to its electrophilic character. Medicinal chemistry investigations have shown that helenalin covalently modifies cysteine 38 and potentially cysteine 120 in the p65 subunit of NF-κB [90], thereby obstructing DNA binding and inhibiting transcription of NF-κB-dependent genes. Direct inhibition of NF-κB is considered advantageous due to the involvement of upstream enzymes in multiple signaling pathways, and targeting p65 may reduce off-target effects. Limited natural availability prompted the synthesis of simplified analogs lacking the seven-membered ring, which retained biological activity and were validated through luciferase reporter assays and proteomic labeling studies. These findings highlight the potential of structurally simplified natural product analogs to maintain potency and selectivity through covalent engagement, and additional analogs have been developed to explore alternative pharmacophores for NF-κB inhibition [52]. Thapsigargin [91,92], derived from *Thapsia garganica* [93], a plant native to Europe, represents a guaianolide with a highly oxidized structure. Unlike other sesquiterpene lactones, it lacks the reactive α-exo-methylene group but features an electrophilic angelate side chain. Thapsigargin irreversibly inhibits the sarco/endoplasmic reticulum calcium ATPases (SERCA) [94,95], leading to calcium leakage, activation of caspases, release of apoptotic mediators, and DNA fragmentation by calcium-dependent endonucleases [52]. This inhibition occurs with high specificity, making thapsigargin a valuable tool for studying calcium dynamics (Table 3). Although its binding mode remains unresolved, its potency has led to the development of mipsagargin [96,97], a PSMA-activated prodrug currently under clinical evaluation for PSMA-positive tumors [98]. Despite its structural complexity, recent synthetic advances have enabled scalable production [52]. Further structural diversification of sesquiterpenes has resulted in oligomeric natural products. In particular, ainsliadimer A, which was previously isolated from *Ainsliaea* species [99], was synthesized and evaluated by the Lei group that identified this molecule as a potent NF-κB inhibitor that selectively binds an allosteric pocket in IKKα and IKKβ through covalent interaction with cysteine 46. A biotinylated version of the compound confirmed target specificity in pull-down assays, distinguishing it as the first known allosteric covalent kinase inhibitor. In contrast, ainsliatrimer A exhibited high selectivity as a PPARγ antagonist, with covalent binding confirmed through knock-down experiments and probe-based assays. These examples illustrate how minor structural modifications can influence preorganization of noncovalent complexes and enhance target selectivity through covalent interactions [52]. Eudesmanolides [100] form a distinct subgroup within the sesquiterpene lactone family. IJ-5, characterized by a 6,6,5 tricyclic framework and an α-exo-methylene moiety, has been identified as the principal bioactive compound in extracts of *Inula japonica*, a medicinal plant traditionally used in Chinese medicine [101] to treat inflammatory conditions such as bronchitis, arthritis, and hepatitis. Recent findings have demonstrated that IJ-5 interferes with the NF-κB signaling cascade. To clarify its molecular target and mechanism of action, IJ-5 was immobilized on sepharose beads and subjected to pull-down and proteomic analyses, which revealed covalent interaction with UbcH5c, a ubiquitin-conjugating enzyme, through modification of the active site cysteine 85. This enzyme belongs to a larger family of E2 enzymes and plays a role in NF-κB pathway regulation. IJ-5 was shown to suppress TNFα-induced activation of this pathway, although it did not exhibit activity against other kinases involved in the same signaling cascade, including IKKα and IKKβ, despite structural similarities with other α-exo-methylene-containing compounds such as ainsliadimer A. Heliangolides [102] represent another prominent class of sesquiterpene lactones. EM23, isolated from *Elephantopus mollis*, a plant used in traditional Chinese medicine [103] for various ailments, has demonstrated anticancer activity. Investigations into its cytotoxic mechanism in chronic myeloid leukemia K562 cells and acute myeloid leukemia HL-60 cells revealed induction of apoptosis through caspase-mediated PARP cleavage and DNA fragmentation [52]. A notable increase in reactive oxygen species was observed following EM23 treatment, and pre-exposure to N-acetylcysteine reversed the apoptotic effects, suggesting a redox-dependent mechanism. Down-regulation of thioredoxin and thioredoxin reductase, key components of the cellular redox system, was also reported. Thioredoxin reductase contains two redox-active residues at its C-terminal region, cysteine 497 and selenocysteine 498, and mass spectrometry combined with pH-dependent analysis confirmed covalent modification at the selenocysteine site. Inhibition of the thioredoxin system resulted in elevated intracellular oxidative stress, increased phosphorylation of ASK1, and activation of downstream pro-apoptotic MAPK kinases including JNK and p38, culminating in programmed cell death. Given the role of the thioredoxin system in modulating NF-κB signaling, suppression of this pathway by EM23 was proposed to contribute to the blockade of TNFα-induced anti-apoptotic responses [52]. In conclusion, plant-derived covalent inhibitors exemplify the therapeutic potential of electrophilic natural products, combining specificity with potent biological effects. Compounds such as isothiocyanates, sesquiterpene lactones, and guaianolides demonstrate versatile mechanisms, including covalent modification of cysteine or selenocysteine residues in key regulatory proteins. Unlike covalent inhibitors derived from fungi, marine organisms, and bacteria, which have been extensively applied in combating pathogenic diseases, plant-based covalent inhibitors have primarily garnered attention for their roles in anticancer and anti-inflammatory therapies. However, computational investigations have increasingly highlighted the antiviral potential of bioactive compounds from medicinal plants. Based on these insights, this review proposes that their utility in antibacterial and broader antipathogenic strategies warrants further exploration, as such applications remain underrepresented despite promising molecular profiles. However, challenges such as bioavailability, selectivity, and the complex equilibria governing covalent interactions remain important considerations for their pharmacological application. Despite these challenges, continued research on these natural scaffolds offers promising avenues for the design of novel, targeted therapeutics.

## 3. Conclusions

Nature continues to serve as an unparalleled reservoir of bioactive compounds, offering structurally diverse and mechanistically rich scaffolds for therapeutic innovation. Among these, covalent inhibitors derived from natural sources, particularly plants, fungi, marine organisms, and microbes, have emerged as a compelling class of molecules capable of irreversibly modulating pathogen-specific enzymes. This review has described the multifaceted potential of such inhibitors, emphasizing their ability to form stable covalent bonds with residues found in target proteins, thereby achieving sustained biological activity and high specificity. Covalent inhibition represents a distinct pharmacological strategy that complements the more transient and reversible interactions of non-covalent inhibitors. While covalent inhibitors offer prolonged target engagement and reduced dosing frequency, they also pose challenges related to off-target reactivity, toxicity, and bioavailability. In fact, caution should be exercised when developing new strategies involving phytocompounds for enzyme inhibition. Certain plant-derived covalent modifiers can be toxic to humans. For example, urushiol, an oily mixture of catecholic compounds produced by plants such as poison ivy (*Toxicodendron radicans*), is readily oxidized in vivo to form electrophilic ortho-quinones that react covalently with nucleophilic amines and thiols on membrane proteins [104]. The haptenization of host proteins by urushiol quinones triggers activation of the immune system, leading to the characteristic allergic contact dermatitis observed at sites of exposure [104]. Nonetheless, natural products have demonstrated an exceptional ability to balance potency with selectivity, as seen in compounds like isothiocyanates, sesquiterpene lactones, guaianolides, fimbrolides, and microbial antibiotics. These molecules not only exhibit therapeutic efficacy across a range of diseases, including infectious diseases, cancer, inflammatory disorders, and neurological conditions, but also provide valuable mechanistic insights into enzyme regulation and inhibition. The examples discussed demonstrate the versatility of covalent inhibition across diverse enzyme classes and biological pathways. From targeting proteases and kinases to modulating transcription factors and redox enzymes, nature-inspired inhibitors have shown remarkable adaptability. Moreover, advances in virtual screening, molecular docking, and structure-based drug design have enabled the rational optimization of these natural scaffolds, enhancing their pharmacokinetic profiles and therapeutic indices. Looking ahead, the future of covalent inhibitor development lies in the integration of natural product chemistry with cutting-edge technologies in medicinal chemistry, computational modeling, and delivery systems. Future efforts will focus on refining selectivity, minimizing adverse effects, and overcoming resistance mechanisms that threaten long-term efficacy. Remarkably, the exploration of underutilized natural sources, such as rare fungi and deep-sea organisms, may yield novel scaffolds with unprecedented biological activity. In conclusion, nature-inspired covalent inhibitors represent a powerful and promising avenue in modern drug discovery thanks to their ability to irreversibly target pathogen enzymes with high specificity, which positions them as valuable tools in the fight against emerging and persistent diseases.

## Figures and Tables

**Table 1:** Comparison between covalent and non-covalent enzyme inhibition.

Feature	Covalent Enzyme Inhibition	Non-Covalent Enzyme Inhibition
Nature of interaction	Forms a stable chemical (covalent) bond with the target enzyme.	Involves reversible physical interactions such as hydrogen bonds, ionic forces, van der Waals interactions, and hydrophobic effects.
Reversibility	Generally irreversible or slowly reversible, depending on the bond stability.	Reversible, allowing the inhibitor to dissociate under physiological conditions.
Target engagement	Provides prolonged and sustained inhibition due to covalent linkage.	Results in transient and dynamic inhibition due to weaker, reversible binding.
Selectivity	Can achieve high selectivity by targeting rare amino acid residues (e.g., cysteine, serine).	Selectivity depends on binding affinity and complementarity of the enzyme–inhibitor interface.
Toxicity and Safety	May cause irreversible drug-induced toxicity or off-target covalent binding.	Generally exhibits a better safety profile due to reversibility.
Pharmacodynamics	Often leads to longer duration of action and reduced dosing frequency.	Allows more tunable pharmacodynamics and reversible modulation of enzyme activity.
Therapeutic Applications	Used in antiviral, anticancer, and anti-inflammatory drug design.	Used in kinase inhibitors, enzyme regulators, and protease inhibitors with reversible binding modes.
Advantages	High potency, durable target engagement, potential for overcoming drug resistance.	Reversible action, lower risk of permanent toxicity, greater control of dose–response.
Limitations	Risk of off-target reactivity, limited reversibility, possible mechanism-related toxicity.	Shorter duration of inhibition, may require higher or more frequent dosing.

**Table 2:** Summary of the main covalent inhibitors derived from fungi, marine organisms, and microbes discussed in this section.

Source	Representative Compound	Reactive Functional Group	Primary Molecular Target	Mechanism/ Type of Covalent Interaction	Biological/Therapeutic Relevance
Fungi	4-Isoavenaciolide	Exo-methylene (Michael acceptor)	VHR phosphatase (Cys124, Cys171)	Michael addition forming covalent adducts	Selective inhibition of dual-specificity and tyrosine phosphatases
Fungi	Hypothemycin (resorcylic acid lactone)	cis-Enone within macrocycle	ERK2 (Cys164) and kinases with CDXG motif	Covalent modification at conserved cysteine residues	Antiparasitic (*Trypanosoma brucei*), antitumor, kinase inhibitor
Fungi	Pyranonaphthoquinone lactones (e.g., 7-Deoxykalafungin)	Quinone and lactone moieties	Akt kinase (Cys310)	Thiol-mediated reduction forming reactive quinone methide intermediate	Potent and selective Akt inhibition (anticancer)
Fungi (*Bipolaris* spp.)	Ophiobolin A	1,4-Dicarbonyl group	Phosphatidylethanolamine (lipid target)	Paal–Knorr pyrrole formation with lysine/amine groups	Paraptosis-like cytotoxicity, membrane disruption in cancer cells
Marine red algae (*Delisea pulchra*)	Fimbrolides	Polybrominated butenolide (bromoolefin)	LuxS (Cys83), LuxE (Cys362), and other quorum-sensing enzymes	Addition–elimination mechanism forming stable covalent adducts	Inhibition of quorum sensing and bacterial communication
Bacteria (*Streptomyces* spp.)	β-Lactam antibiotics (e.g., penicillin, carbapenems)	β-Lactam ring	Penicillin-binding proteins (serine transpeptidases)	Acylation of catalytic serine residue, irreversible inhibition	Bactericidal; inhibition of cell wall synthesis
Bacteria (*Streptomyces clavuligerus*)	Clavulanic acid	β-Lactam	Serine β-lactamases	Suicide inhibition via irreversible covalent binding	β-Lactamase inhibitor enhancing antibiotic efficacy; neuroprotective potential
Bacteria (*Streptomyces fradiae*)	Fosfomycin	Epoxide ring	MurA enzyme (Cys115)	Nucleophilic attack on epoxide; irreversible inactivation	Inhibition of peptidoglycan biosynthesis; broad-spectrum antibiotic
Bacteria (modified by gut microbiota)	Menadione (Vitamin K_3_)	Quinone moiety	CDC25A phosphatase (active-site cysteine)	Covalent modification of catalytic cysteine	Antiproliferative; potential anticancer activity

**Table 3:** Summary of the main covalent inhibitors derived from plants mentioned in this section.

Source	Representative Compound	Reactive Functional Group	Primary Molecular Target	Mechanism/Type of Covalent Interaction	Biological/Therapeutic Relevance
Cruciferous vegetables	Allyl isothiocyanate, Sulforaphane, Phenethyl isothiocyanate	Isothiocyanate (-N=C=S)	Cysteine and lysine residues; TRPA1 ion channel	Covalent modification; reversible glutathione conjugation	Cancer chemoprevention; neurological disorders (schizophrenia, autism)
Feverfew (*Tanacetum parthenium*)	Parthenolide	α-exo-methylene-γ-butyrolactone, epoxide	IKKβ (CYS179), NF-κB signaling	Covalent modification of cysteine	Anti-inflammatory; acute myelogenous leukemia; drug-resistant glioblastoma
*Elephantopus scaber*	Deoxyelephantopin	Germacranolide	PPARγ (CYS176); proteasome	Covalent binding in zinc-finger domain	Anticancer (breast tumors); apoptosis induction
*Arnica*, *Helenium* species	Helenalin	α-exo-methylene-γ-butyrolactone, cyclopentenone	NF-κB p65 subunit (CYS38, CYS120)	Covalent cysteine modification	Anti-inflammatory; inhibition of NF-κB-dependent gene transcription
*Thapsia garganica*	Thapsigargin	Electrophilic angelate side chain	SERCA (calcium ATPases)	Irreversible covalent inhibition	Apoptosis induction; tool for cancer research; PSMA-targeted prodrug mipsagargin
*Ainsliaea* species	Ainsliadimer A	α-exo-methylene	IKKα/β (CYS46), PPARγ	Covalent modification at allosteric site	NF-κB inhibition; selective kinase and nuclear receptor targeting
*Inula japonica*	IJ-5 (Eudesmanolide)	α-exo-methylene	UbcH5c (CYS85), NF-κB pathway	Covalent modification of active site cysteine	Anti-inflammatory; selective inhibition of ubiquitin-conjugating enzyme
*Elephantopus mollis*	EM23 (Heliangolide)	α-exo-methylene	Thioredoxin reductase (CYS497, Sec498)	Covalent modification of selenocysteine	Anticancer; redox-mediated apoptosis; suppression of NF-κB signaling

## Data Availability

No new data were created or analyzed in this study.

## Abbreviations

The following abbreviations are used in this manuscript:

Akt	Protein kinase B
ASK1	Apoptosis signal-regulating kinase 1
ATPase	Adenosine triphosphatase
CDC25A	Cell division cycle 25A phosphatase
Cys	Cysteine
EM23	Heliangolide from Elephantopus mollis
ERK2	Extracellular signal-regulated kinase 2
GLT-1	Glutamate transporter 1
IKKα	IκB kinase alpha
IKKβ	IκB kinase beta
IJ-5	Eudesmanolide from *Inula japonica*
LC-1	Parthenolide prodrug with enhanced solubility
Lys	Lysine
LuxE	Acyl-protein synthetase in quorum-sensing pathway
LuxS	S-ribosylhomocysteine lyase
MAPK	Mitogen-activated protein kinase
MPC-1	Modified penicillin compound for enhanced β-lactamase inhibition
MurA	UDP-N-acetylglucosamine-enolpyruvyltransferase
NAC	N-acetylcysteine
NF-κB	Nuclear factor kappa-light-chain-enhancer of activated B cells
PARP	Poly (ADP-ribose) polymerase
PBP	Penicillin-binding protein
PHA B	Polyhydroxybutyrate biosynthesis enzyme
PKAα	Protein kinase A alpha
PP1	Protein phosphatase 1
PP2A	Protein phosphatase 2A
PPARγ	Peroxisome proliferator-activated receptor gamma
PSMA	Prostate-specific membrane antigen
SERCA	Sarco/endoplasmic reticulum calcium ATPase
TRPA1	Transient receptor potential ankyrin 1

## References

[1] Robertson J.G. (2005). Mechanistic basis of enzyme-targeted drugs. Biochemistry.

[2] Griffith D., P Parker J., J Marmion C. (2010). Enzyme inhibition as a key target for the development of novel metal-based anti-cancer therapeutics. Anti-Cancer Agents Med. Chem. (Former. Curr. Med. Chem.-Anti-Cancer Agents).

[3] Flury P., Vishwakarma J., Sylvester K., Higashi-Kuwata N., Dabrowska A.K., Delgado R., Cuell A., Basu R., Taylor A.B., de Oliveira E.G. (2025). Azapeptide-Based SARS-CoV-2 Main Protease Inhibitors: Design, Synthesis, Enzyme Inhibition, Structural Determination, and Antiviral Activity. J. Med. Chem..

[4] Rotschafer J.C., Ostergaard B.E. (1995). Combination β-lactam and β-lactamase-inhibitor products: Antimicrobial activity and efficiency of enzyme inhibition. Am. J. Health-Syst. Pharm..

[5] Schaefer D., Cheng X. (2023). Recent advances in covalent drug discovery. Pharmaceuticals.

[6] Kim G., Grams R.J., Hsu K.-L. (2025). Advancing Covalent Ligand and Drug Discovery beyond Cysteine. Chem. Rev..

[7] Faridoon, Zheng J., Zhang G., Li J.J. (2025). Key Advances in the Development of Reversible Covalent Inhibitors.

[8] Wu G.-Y., Xiao M.-Z., Hao W.-C., Yang Z.-S., Liu X.-R., Xu D.-S., Peng Z.-X., Zhang L.-Y. (2025). Drug resistance in breast cancer: Mechanisms and strategies for management. Drug Resist. Updates.

[9] Vicidomini C., Roviello G.N. (2025). Therapeutic Convergence in Neurodegeneration: Natural Products, Drug Repurposing, and Biomolecular Targets. Biomolecules.

[10] Costanzo M., Roviello G.N. (2025). Precision Therapeutics Through Bioactive Compounds: Metabolic Reprogramming, Omics Integration, and Drug Repurposing Strategies. Int. J. Mol. Sci..

[11] Mittova V., Pirtskhalava M., Tsetskhladze Z.R., Makalatia K., Loladze A., Bebiashvili I., Barblishvili T., Gogoladze A., Roviello G.N. (2025). Antioxidant Potential and Antibacterial Activities of Caucasian Endemic Plants *Sempervivum transcaucasicum* and *Paeonia daurica* subsp. *mlokosewitschii* Extracts and Molecular In Silico Mechanism Insights. J. Xenobiotics.

[12] Falanga A.P., Piccialli I., Greco F., D’Errico S., Nolli M.G., Borbone N., Oliviero G., Roviello G.N. (2025). Nanostructural Modulation of G-Quadruplex DNA in Neurodegeneration: Orotate Interaction Revealed Through Experimental and Computational Approaches. J. Neurochem..

[13] Sargsyan T., Simonyan H.M., Stepanyan L., Tsaturyan A., Vicidomini C., Pastore R., Guerra G., Roviello G.N. (2025). Neuroprotective Properties of Clove (*Syzygium aromaticum*): State of the Art and Future Pharmaceutical Applications for Alzheimer’s Disease. Biomolecules.

[14] Mugisa S., Murahari M. (2025). Covalent Inhibitors-An Overview of Design Process, Challenges and Future Directions. Curr. Org. Chem..

[15] Hu Q., Zhang Y.-W., Zhang Y.-N., Zhu G.-H., Chen P.-C., Liu W., Hu X.-P., Song F.-F., Pan Z.-F., Zheng S.-L. (2025). Uncovering the naturally occurring covalent inhibitors of SARS-CoV-2 Mpro from the Chinese medicine sappanwood and deciphering their synergistic anti-Mpro effects. J. Ethnopharmacol..

[16] Curieses Andrés C.M., Lobo F., Pérez de Lastra J.M., Bustamante Munguira E., Andrés Juan C., Pérez-Lebeña E. (2025). Cysteine Alkylation in Enzymes and Transcription Factors: A Therapeutic Strategy for Cancer. Cancers.

[17] Patel D., Huma Z.E., Duncan D. (2024). Reversible Covalent Inhibition—Desired Covalent Adduct Formation by Mass Action. ACS Chem. Biol..

[18] Sanaullah B., Truong N.V., Nguyen T.-K., Han E.-T. (2025). Combating malaria: Targeting the ubiquitin-proteasome system to conquer drug resistance. Trop. Med. Infect. Dis..

[19] Tsai C.-H., Lee P.-Y., Stollar V., Li M.-L. (2006). Antiviral therapy targeting viral polymerase. Curr. Pharm. Des..

[20] Biancheri P., Di Sabatino A., Corazza G.R., MacDonald T.T. (2013). Proteases and the gut barrier. Cell Tissue Res..

[21] Samuels S., Taillandier D., Aurousseau E., Cherel Y., Le Maho Y., Arnal M., Attaix D. (1996). Gastrointestinal tract protein synthesis and mRNA levels for proteolytic systems in adult fasted rats. Am. J. Physiol.-Endocrinol. Metab..

[22] Vergnolle N. (2016). Protease inhibition as new therapeutic strategy for GI diseases. Gut.

[23] Macedo M.L.R., de Oliveira C.F.R., Costa P.M., Castelhano E.C., Silva-Filho M.C. (2015). Adaptive mechanisms of insect pests against plant protease inhibitors and future prospects related to crop protection: A review. Protein Pept. Lett..

[24] Moloi S.J., Ngara R. (2023). The roles of plant proteases and protease inhibitors in drought response: A review. Front. Plant Sci..

[25] Sabotič J., Kos J. (2012). Microbial and fungal protease inhibitors—Current and potential applications. Appl. Microbiol. Biotechnol..

[26] Upadhyay A., Pal D., Kumar A. (2024). Combinatorial therapeutic enzymes to combat multidrug resistance in bacteria. Life Sci..

[27] Urban-Chmiel R., Marek A., Stępień-Pyśniak D., Wieczorek K., Dec M., Nowaczek A., Osek J. (2022). Antibiotic resistance in bacteria—A review. Antibiotics.

[28] Clatworthy A.E., Pierson E., Hung D.T. (2007). Targeting virulence: A new paradigm for antimicrobial therapy. Nat. Chem. Biol..

[29] Yu Y., Wang Z., Chai Z., Ma S., Li A., Li Y. (2025). Central Nervous System-Derived Extracellular Vesicles as Biomarkers in Alzheimer’s Disease. Int. J. Mol. Sci..

[30] De Strooper B., Vassar R., Golde T. (2010). The secretases: Enzymes with therapeutic potential in Alzheimer disease. Nat. Rev. Neurol..

[31] Renzi G., Carta F., Supuran C.T. (2023). The integrase: An overview of a key player enzyme in the antiviral scenario. Int. J. Mol. Sci..

[32] Nair V., Chi G. (2007). HIV integrase inhibitors as therapeutic agents in AIDS. Rev. Med. Virol..

[33] Chandra K., Das P., Mamidi S., Hurevich M., Iosub-Amir A., Metanis N., Reches M., Friedler A. (2016). Covalent Inhibition of HIV-1 Integrase by N-Succinimidyl Peptides. ChemMedChem.

[34] Zhang S., Xue X., Zhang L., Zhang L., Liu Z. (2015). Comparative Analysis of Pharmacophore Features and Quantitative Structure–Activity Relationships for CD 38 Covalent and Non-covalent Inhibitors. Chem. Biol. Drug Des..

[35] Aljoundi A., Bjij I., El Rashedy A., Soliman M.E. (2020). Covalent versus non-covalent enzyme inhibition: Which route should we take? A justification of the good and bad from molecular modelling perspective. Protein J..

[36] Groll M., Huber R. (2003). Substrate access and processing by the 20S proteasome core particle. Int. J. Biochem. Cell Biol..

[37] Scott K., Hayden P.J., Will A., Wheatley K., Coyne I. (2016). Bortezomib for the treatment of multiple myeloma. Cochrane Database Syst. Rev..

[38] Beck P., Dubiella C., Groll M. (2012). Covalent and non-covalent reversible proteasome inhibition. Biol. Chem..

[39] Teicher B.A., Tomaszewski J.E. (2015). Proteasome inhibitors. Biochem. Pharmacol..

[40] Akher F.B., Farrokhzadeh A., Soliman M.E. (2019). Covalent vs. Non-Covalent Inhibition: Tackling Drug Resistance in EGFR–A Thorough Dynamic Perspective. Chem. Biodivers..

[41] Burger J.A. (2019). Bruton tyrosine kinase inhibitors: Present and future. Cancer J..

[42] Shadman M., Burke J.M., Cultrera J., Yimer H.A., Zafar S.F., Misleh J., Rao S.S., Farber C.M., Cohen A., Yao H. (2025). Zanubrutinib is well tolerated and effective in patients with CLL/SLL intolerant of ibrutinib/acalabrutinib: Updated results. Blood Adv..

[43] Zain R., Vihinen M. (2021). Structure-function relationships of covalent and non-covalent BTK inhibitors. Front. Immunol..

[44] Shi Y., Wang G., Cai X.-p., Deng J.-w., Zheng L., Zhu H.-h., Zheng M., Yang B., Chen Z. (2020). An overview of COVID-19. J. Zhejiang Univ.-Sci. B.

[45] Bar-On Y.M., Flamholz A., Phillips R., Milo R. (2020). SARS-CoV-2 (COVID-19) by the numbers. Elife.

[46] Agost-Beltrán L., de la Hoz-Rodríguez S., Bou-Iserte L., Rodríguez S., Fernández-de-la-Pradilla A., González F.V. (2022). Advances in the development of SARS-CoV-2 Mpro inhibitors. Molecules.

[47] Awoonor-Williams E., Abu-Saleh A.A.-A.A. (2021). Covalent and non-covalent binding free energy calculations for peptidomimetic inhibitors of SARS-CoV-2 main protease. Phys. Chem. Chem. Phys..

[48] Jia C., Chai J., Zhang S., Sun Y., He L., Sang Z., Chen D., Zheng X. (2025). The advancements of marine natural products in the treatment of alzheimer’s disease: A study based on cell and animal experiments. Mar. Drugs.

[49] Zhai X.-Y., Liu J.-J., Wang C.-D., Dou Y.-F., Lv J.-H., Wang L.-A., Zhang J.-X., Li Z. (2025). Novel polycyclic meroterpenoids with protein tyrosine phosphatase 1B inhibitory activity isolated from desert-derived fungi Talaromyces sp. HMT-8. Nat. Prod. Bioprospecting.

[50] Gallorini M., Carradori S., Panieri E., Sova M., Saso L. (2024). Modulation of NRF2: Biological dualism in cancer, targets and possible therapeutic applications. Antioxid. Redox Signal..

[51] Ruddraraju K.V., Zhang Z.-Y. (2017). Covalent inhibition of protein tyrosine phosphatases. Mol. Biosyst..

[52] Lagoutte R., Winssinger N. (2017). Following the lead from nature with covalent inhibitors. Chimia.

[53] Nishino M., Choy J.W., Gushwa N.N., Oses-Prieto J.A., Koupparis K., Burlingame A.L., Renslo A.R., McKerrow J.H., Taunton J. (2013). Hypothemycin, a fungal natural product, identifies therapeutic targets in Trypanosoma brucei. Elife.

[54] Salaski E.J., Krishnamurthy G., Ding W.-D., Yu K., Insaf S.S., Eid C., Shim J., Levin J.I., Tabei K., Toral-Barza L. (2009). Pyranonaphthoquinone lactones: A new class of AKT selective kinase inhibitors alkylate a regulatory loop cysteine. J. Med. Chem..

[55] Tsuna K., Noguchi N., Nakada M. (2011). Convergent total synthesis of (+)-ophiobolin A. Angew. Chem.-Int. Ed..

[56] Kornienko A., La Clair J.J. (2017). Covalent modification of biological targets with natural products through Paal–Knorr pyrrole formation. Nat. Prod. Rep..

[57] Lowery C.A., Abe T., Park J., Eubanks L.M., Sawada D., Kaufmann G.F., Janda K.D. (2009). Revisiting AI-2 quorum sensing inhibitors: Direct comparison of alkyl-DPD analogues and a natural product fimbrolide. J. Am. Chem. Soc..

[58] Pei D., Zhu J. (2004). Mechanism of action of S-ribosylhomocysteinase (LuxS). Curr. Opin. Chem. Biol..

[59] Zhu J., Dizin E., Hu X., Wavreille A.-S., Park J., Pei D. (2003). S-Ribosylhomocysteinase (LuxS) is a mononuclear iron protein. Biochemistry.

[60] Tian Q., Wu J., Xu H., Hu Z., Huo Y., Wang L. (2022). Cryo-EM structure of the fatty acid reductase LuxC–LuxE complex provides insights into bacterial bioluminescence. J. Biol. Chem..

[61] Kim J., Chang J.H., Kim E.-J., Kim K.-J. (2014). Crystal structure of (R)-3-hydroxybutyryl-CoA dehydrogenase PhaB from *Ralstonia eutropha*. Biochem. Biophys. Res. Commun..

[62] Qi Q., Rehm B.H. (2001). Polyhydroxybutyrate biosynthesis in Caulobacter crescentus: Molecular characterization of the polyhydroxybutyrate synthase. Microbiology.

[63] Papp-Wallace K.M., Endimiani A., Taracila M.A., Bonomo R.A. (2011). Carbapenems: Past, present, and future. Antimicrob. Agents Chemother..

[64] Breilh D., Texier-Maugein J., Allaouchiche B., Saux M.-C., Boselli E. (2013). Carbapenems. J. Chemother..

[65] Li R., Lloyd E.P., Moshos K.A., Townsend C.A. (2014). Identification and characterization of the carbapenem MM 4550 and its gene cluster in Streptomyces argenteolus ATCC 11009. ChemBioChem.

[66] Ri Y.J., Ri C.H., Ho U.H., Song S.R., Pak I.S., Ri T.R., Kim Y.J., Ri J.S. (2025). Association between intraspecific variability and penicillin production in industrial strain, *Penicillium chrysogenum* revealed by RAPD and SRAP markers. World J. Microbiol. Biotechnol..

[67] Lee M., Hesek D., Suvorov M., Lee W., Vakulenko S., Mobashery S. (2003). A mechanism-based inhibitor targeting the DD-transpeptidase activity of bacterial penicillin-binding proteins. J. Am. Chem. Soc..

[68] Pan X., He Y., Chen T., Chan K.-F., Zhao Y. (2017). Modified penicillin molecule with carbapenem-like stereochemistry specifically inhibits class C β-lactamases. Antimicrob. Agents Chemother..

[69] Liu L., Chen Z., Liu W., Ke X., Tian X., Chu J. (2022). Cephalosporin C biosynthesis and fermentation in *Acremonium chrysogenum*. Appl. Microbiol. Biotechnol..

[70] Tollnick C., Seidel G., Beyer M., Schügerl K. (2003). Investigations of the production of cephalosporin C by *Acremonium chrysogenum*. New Trends and Developments in Biochemical Engineering.

[71] Ball A., Davey P., Geddes A., Farrell I., Brookes G. (1980). Clavulanic acid and amoxycillin: A clinical, bacteriological, and pharmacological study. Lancet.

[72] Shoae-Hagh P., Razavi B.M., Sadeghnia H.R., Mehri S., Karimi G., Hosseinzadeh H. (2025). Molecular and Behavioral Neuroprotective Effects of Clavulanic Acid and Crocin in Haloperidol-Induced Tardive Dyskinesia in Rats. Mol. Neurobiol..

[73] Gómez-Ríos D., Gómez-Gaona L.M., Ramírez-Malule H. (2024). Clavulanic Acid Overproduction: A Review of Environmental Conditions, Metabolic Fluxes, and Strain Engineering in Streptomyces clavuligerus. Fermentation.

[74] Balcazar-Ochoa L.G., Ventura-Martínez R., Ángeles-López G.E., Gómez-Acevedo C., Carrasco O.F., Sampieri-Cabrera R., Chavarría A., González-Hernández A. (2024). Clavulanic acid and its potential therapeutic effects on the central nervous system. Arch. Med. Res..

[75] Rothstein J.D., Patel S., Regan M.R., Haenggeli C., Huang Y.H., Bergles D.E., Jin L., Dykes Hoberg M., Vidensky S., Chung D.S. (2005). β-Lactam antibiotics offer neuroprotection by increasing glutamate transporter expression. Nature.

[76] Higgins L.J., Yan F., Liu P., Liu H.-w., Drennan C.L. (2005). Structural insight into antibiotic fosfomycin biosynthesis by a mononuclear iron enzyme. Nature.

[77] Bergogne-Bérézin E. (2005). Fosfomycin and derivatives. Antimicrobial Agents: Antibacterials and Antifungals.

[78] Silver L.L. (2017). Fosfomycin: Mechanism and resistance. Cold Spring Harb. Perspect. Med..

[79] Altwiley D., Brignoli T., Edwards A., Recker M., Lee J.C., Massey R.C. (2021). A functional menadione biosynthesis pathway is required for capsule production by Staphylococcus aureus. Microbiology.

[80] Shearer M.J. (2022). The biosynthesis of menaquinone-4: How a historic biochemical pathway is changing our understanding of vitamin K nutrition. J. Nutr..

[81] Beulens J.W., Booth S.L., van den Heuvel E.G., Stoecklin E., Baka A., Vermeer C. (2013). The role of menaquinones (vitamin K2) in human health. Br. J. Nutr..

[82] Conly J., Stein K. (1992). The production of menaquinones (vitamin K2) by intestinal bacteria and their role in maintaining coagulation homeostasis. Prog. Food Nutr. Sci..

[83] Marchionatti A.M., Picotto G., Narvaez C.J., Welsh J., de Talamoni N.G.T. (2009). Antiproliferative action of menadione and 1, 25 (OH) 2D3 on breast cancer cells. J. Steroid Biochem. Mol. Biol..

[84] Gehringer M., Laufer S.A. (2018). Emerging and re-emerging warheads for targeted covalent inhibitors: Applications in medicinal chemistry and chemical biology. J. Med. Chem..

[85] Bakheit A.H., Saquib Q., Ahmed S., Ansari S.M., Al-Salem A.M., Al-Khedhairy A.A. (2023). Covalent inhibitors from saudi medicinal plants target RNA-dependent RNA polymerase (RdRp) of SARS-CoV-2. Viruses.

[86] Zarei E., Zarei A., Omidkhoda A. (2025). Parthenolide: Pioneering new frontiers in hematological malignancies. Front. Pharmacol..

[87] Lehrke M., Lazar M.A. (2005). The many faces of PPARγ. Cell.

[88] Ye J. (2008). Regulation of PPARγ function by TNF-α. Biochem. Biophys. Res. Commun..

[89] Ng D., Altamirano-Vallejo J.C., Navarro-Partida J., Sanchez-Aguilar O.E., Inzunza A., Valdez-Garcia J.E., Gonzalez-de-la-Rosa A., Bustamante-Arias A., Armendariz-Borunda J., Santos A. (2024). Enhancing Ocular Surface in Dry Eye Disease Patients: A Clinical Evaluation of a Topical Formulation Containing Sesquiterpene Lactone Helenalin. Pharmaceuticals.

[90] Sizemore N., Lerner N., Dombrowski N., Sakurai H., Stark G.R. (2002). Distinct roles of the IκB kinase α and β subunits in liberating nuclear factor κB (NF-κB) from IκB and in phosphorylating the p65 subunit of NF-κB. J. Biol. Chem..

[91] Khurram I., Khan M.U., Ibrahim S., Ghani M.U., Amin I., Falzone L., Herrera-Bravo J., Setzer W.N., Sharifi-Rad J., Calina D. (2024). Thapsigargin and its prodrug derivatives: Exploring novel approaches for targeted cancer therapy through calcium signaling disruption. Med. Oncol..

[92] Suresh A., Bagchi D., Kaliappan K.P. (2024). Thapsigargin: A promising natural product with diverse medicinal potential-a review of synthetic approaches and total syntheses. Org. Biomol. Chem..

[93] Makunga N.P., Jäger A.K., van Staden J. (2005). An improved system for the in vitro regeneration of Thapsia garganica via direct organogenesis–influence of auxins and cytokinins. Plant Cell Tissue Organ Cult..

[94] de Mello M.M.B., de Oliveira Neves V.G., Parente J.M., Pernomian L., de Oliveira I.S., Pedersoli C.A., Awata W.M.C., Tirapelli C.R., Arantes E.C., Tostes R.d.C.A. (2024). Sarcoplasmic reticulum calcium ATPase (SERCA) proteolysis by matrix metalloproteinase-2 contributes to vascular dysfunction in early hypertension. Eur. J. Pharmacol..

[95] Yu Q., Tian W. (2025). The role of SERCA in vascular diseases, a potential therapeutic target. Cell Calcium.

[96] Andersen T.B., López C.Q., Manczak T., Martinez K., Simonsen H.T. (2015). Thapsigargin—From *Thapsia* L. to mipsagargin. Molecules.

[97] Isaacs J.T., Brennen W.N., Christensen S.B., Denmeade S.R. (2021). Mipsagargin: The beginning—Not the end—Of thapsigargin prodrug-based cancer therapeutics. Molecules.

[98] Piccioni D., Juarez T., Brown B., Rose L., Allgood V., Kesari S. (2015). Atct-18: Phase II Study of Mipsagargin (G-202), A Psma-Activated Prodrug Targeting the Tumor Endothelium, in Adult Patients with Recurrent or Progressive Glioblastoma. Neuro-Oncol..

[99] Wu Z.-J., Xu X.-K., Shen Y.-H., Su J., Tian J.-M., Liang S., Li H.-L., Liu R.-H., Zhang W.-D. (2008). Ainsliadimer A, a new sesquiterpene lactone dimer with an unusual carbon skeleton from Ainsliaea macrocephala. Org. Lett..

[100] Teresa L.D.P., Caballero E., Anaya J., Caballero C., Gonzalez M. (1986). Eudesmanolides from *Chamaemelum fuscatum*. Phytochemistry.

[101] Li J., Guo X., Luo Z., Wu D., Shi X., Xu L., Zhang Q., Xie C., Yang C. (2024). Chemical constituents from the flowers of Inula japonica and their anti-inflammatory activity. J. Ethnopharmacol..

[102] Onoja S.O., Nnadi C.O., Udem S.C., Anaga A.O. (2020). Potential antidiabetic and antioxidant activities of a heliangolide sesquiterpene lactone isolated from *Helianthus annuus* L. leaves. Acta Pharm..

[103] Wang L., Jian S., Nan P., Liu J., Zhong Y. (2005). Chemotypical variability of leaf oils in *Elephantopus scaber* from 12 locations in China. Chem. Nat. Compd..

[104] Bauer R.A. (2015). Covalent inhibitors in drug discovery: From accidental discoveries to avoided liabilities and designed therapies. Drug Discov. Today.

